# A Direct Examination of the Effect of Intranasal Administration of Oxytocin on Approach-Avoidance Motor Responses to Emotional Stimuli

**DOI:** 10.1371/journal.pone.0058113

**Published:** 2013-02-28

**Authors:** Angeliki Theodoridou, Ian S. Penton-Voak, Angela C. Rowe

**Affiliations:** University of Bristol, School of Experimental Psychology, Bristol, United Kingdom; French National Centre for Scientific Research, France

## Abstract

Oxytocin has been shown to promote a host of social behaviors in humans but the exact mechanisms by which it exerts its effects are unspecified. One prominent theory suggests that oxytocin increases approach and decreases avoidance to social stimuli. Another dominant theory posits that oxytocin increases the salience of social stimuli. Herein, we report a direct test of these hypotheses. In a double-blind, placebo-controlled study we examined approach-avoidance motor responses to social and non-social emotional stimuli. One hundred and twenty participants self-administered either 24 IU oxytocin or placebo and moved a lever toward or away from pictures of faces depicting emotional expressions or from natural scenes appearing before them on a computer screen. Lever movements toward stimuli decreased and movements away increased stimuli size producing the illusion that stimuli moved away from or approached participants. Reaction time data were recorded. The task produced the effects that were anticipated on the basis of the approach-avoidance literature in relation to emotional stimuli, yet the anticipated speeded approach and slowed avoidance responses to emotional faces by the oxytocin group were not observed. Interestingly, the oxytocin treatment group was faster to approach and avoid faces depicting disgust relative to the placebo group, suggesting a salience of disgust for the former group. Results also showed that within the oxytocin group women's reaction times to all emotional faces were faster than those of men, suggesting sex specific effects of oxytocin. The present findings provide the first direct evidence that intranasal oxytocin administration does not enhance approach/avoidance to social stimuli and does not exert a stronger effect on social vs. non-social stimuli in the context of processing of emotional expressions and scenes. Instead, our data suggest that oxytocin administration increases the salience of certain social stimuli and point to a possible role for oxytocin in behavioral prophylaxis.

## Introduction

The neuropeptide oxytocin (OT) enhances social cognitive functioning in humans and is important in interpersonal behavior and affiliation [1], [2]. This highly conserved neuropeptide promotes bonding behaviors and attachments, such as the mother-infant bond, in human and non-human mammals [3], [4]. Although most studies to date have focused on the pro-social effects of OT, for example showing that experimental OT elevation increases judgments of attractiveness and trustworthiness of others [5], recent studies suggest it also increases envy and gloating [6], ethnocentrism [7] and in-group love and protectionism [8]. While originally OT was thought to promote human affiliation through enhancing pro-social emotions and judgments – and has even been described by researchers as the ‘love hormone’ [9] – findings showing that it also increases negative inter-personal and inter-group judgments have led to a re-evaluation of its role in social cognition [6].

One theory put forward to explain OT's effects on social cognition suggests that OT simply renders socially relevant stimuli more salient [6]. Some studies of OT's effects on recognition of important social stimuli, such as facial depictions of emotion, show that intranasal OT selectively enhances the recognition of fear and happiness [10], [11], [12] and slows down the recognition of fear [13], while others indicate that OT enhances the identification of emotions regardless of valence [14], relative to placebo. A further study suggests that intranasal OT decreases aversion to angry faces when there is a financial reward for the selection of such faces [15], possibly through dampening amygdala activation in response to fear-inducing stimuli [16], [17], although other studies have shown OT to decrease amygdala activity during the viewing of both positive (happy) and negative (angry, fearful) faces [18] and even to increase reactivity in the amygdala during the processing of fearful faces in females [19]. Overall, these studies tend to support a salience account of OT effects for some emotions regardless of valence.

A more dominant theory of OT's effects on social cognition suggests that it increases approach toward and decreases avoidance of social stimuli [20], [18], [21], [22] but this theory is yet to be directly tested in humans. Humans and other animals ascribe valences to objects in the environment and responses to their valence are manifested in immediate approach and withdrawal to objects and persons [23], [24], [25]. This view accords with neurophysiological analyses of learning mechanisms, such as that of Schneirla [26]. As a general principle individuals are faster to approach stimuli in the environment they perceive as positive (e.g., positive words) than stimuli they perceive as negative (e.g., negative words) and show faster avoidance of negative stimuli relative to positive [27], [28], [29], [30]. Arm flexion (approach) is elicited by happy faces and faster arm extension (avoidance) is elicited by angry faces [31], [32], while faces depicting disgust elicit relatively complex motor responses (i.e., conscious avoidance but no automatic approach/avoidance [33]).

Here, we directly examine whether intranasal administration of OT spray increases approach to social stimuli (faces with different emotional expressions) relative to non-social stimuli that vary in valence (positive and negative scenes). Given the mixed findings regarding OT's effects on the processing of social stimuli of different valences, we predicted that OT spray would enhance approach and decrease avoidance to all emotional expressions relative to placebo treatment. In case differential effects emerged for the different emotional faces along the lines of previously reported findings [11] we included faces depicting five emotions: happiness, sadness, anger, fear and disgust. Furthermore, as OT administration has been shown to enhance recognition of faces but not non-social stimuli [34] and to exert a more pronounced effect on amygdala deactivation while viewing faces vs. non-social scenes [16], we predicted it would elicit faster approach-avoidance responses to social compared to non-social stimuli. Finally, in the absence of substantial direct evidence for sex-specific OT effects, we hypothesized that intranasal OT administration would affect approach-avoidance motor tendencies similarly in men and women.

## Materials and Methods

### Ethics statement

The study protocol was approved by the Faculty of Science Human Research Ethics Committee at the University of Bristol. All participants provided written informed consent form.

### Participants

One hundred and twenty healthy participants were recruited mainly from the university student population in Bristol, UK and were randomly assigned to receive OT (n = 60, 50% male) or Placebo (n = 60, 50% male). Participants' ages ranged from 18.1 to 43.8 years, and the average age was 22.4 years. Fifty two were romantically attached and 68 single. All participants were fluent in English. Key selection criteria were: not pregnant, not trying to become pregnant, not breastfeeding, not on medication, no history of significant psychiatric or medical illness, and no high blood pressure. Baseline questionnaires confirmed that no participant suffered from severe depression (OT: M = 9.60, SD  = 5.55, Placebo: M = 11.61, SD  = 6.55) or trait anxiety (OT: M = 39.05, SD  = 10.53; Placebo: M = 42.71, SD  = 10.23) as assessed by the Major Depression Inventory (MDI [35]) and the State-Trait Anxiety Inventory (STAI [36]), respectively.

### Design

This study employed a double-blind placebo-controlled design. We examined the effect of OT administration on approach and avoidance motor responses to social and non-social stimuli of positive and negative valence. Reaction time (RT) and error rate data were recorded. We also assessed participants' mood and OT's potential sex dimorphic effects. These measures formed part of a larger study that lasted approximately 50 min. The overall session lasted up to 2 hours as participants were required to remain in the laboratory to guard for potential side effects.

### Stimuli

Images of facial and non-facial stimuli were used. The facial stimuli comprised of morphed male faces depicting one of five emotions, namely, fear, anger, sadness, disgust, and happiness. Each emotional face was created from exemplars from the ‘Karolinska Directed Emotional Faces’ set [37] that had been averaged using PsychoMorph software [38]. The faces were presented one at a time in a grey background in the centre of the screen and gazed directly at the observer. Non-facial stimuli depicted positive scenes (e.g. tranquil landscapes) and negative scenes (e.g. a burning house), were pre-rated on valence by 20 postgraduate psychology students, and resized using Adobe Photoshop 6.0.1. The approach-avoidance task was programmed and presented by the use of E-Prime 2.0 (Psychology Software Tools, Inc.), and a joystick (ATK, Logitech) that was connected to the desktop was used to perform the task.

### Task

Stimuli were presented one at a time on a desktop computer screen. Each image appeared and stayed on the screen until a joystick was either pulled towards the self or pushed away from the self. When the joystick was pulled the image became larger (quadrupled in area) creating the impression that the observer approached the image. In contrast, when the joystick was pushed the image became smaller (quartered in area) creating the impression that the observer moved away from the image. The size of each stimulus was 381×381 pixels at its initial frame, 191×191 pixels in its enlarged version, and 762×762 pixels in its reduced version. The instruction was to push towards one affective stimulus and pull away from another. For example, in one condition the instruction was to push the joystick in response to the happy face and pull in response to the angry face, whereas in another condition, the instruction was to push the joystick in response to the angry face and pull in response to the happy face. There were 22 blocks of 12 experimental trials, yielding 264 experimental trials per participant; twenty blocks contained facial stimuli and two contained non-facial stimuli. Each facial block presented a different combination of two of the five emotional faces (e.g., push for fear – pull for sad, push for sad – pull for fear), and each non-facial block presented a different combination of positive and negative non-facial images (e.g., push for positive scenes-pull for negative scenes). As there were 10 possible combinations for the emotional faces and two for the non-facial stimuli, and each stimulus was subject to both push and pull conditions for each combination, there were 22 possible variations. The blocks were presented in a randomized order as were the images within each block. Four practice trials were completed at the start of each block. Before each trial, a fixation cross appeared for 500 ms and each image remained on the screen until a response was made. Participants were instructed to respond as quickly and accurately as possible.

### Procedure

Approximately a week after a baseline session completed at home, participants were invited to the laboratory to individually complete the experimental session. They were instructed to abstain from alcohol, caffeine and nicotine for 24 hours before testing and food and drink, except water, for 2 hours before testing. At the start of the experimental session, participants were randomly allocated to one of two conditions: they either self-administered an intranasal dose of 24 IU OT (Syntocinon Spray, Novartis, 3 puffs per nostril) or placebo. The placebo sprays contained all ingredients in the OT nasal sprays except for OT. Mood (affect, wakefulness, and calmness) were assessed by the use of the short form of the Multidimensional Mood State Questionnaire [39], a 6-point scale consisting of 15 items with answers ranging from ‘definitely not’ to ‘extremely’. This measure was completed at two time points: immediately before drug administration and immediately before testing. Thirty minutes after drug administration participants started completing a battery of tasks that were presented in two blocks in a counterbalanced order. Half the time the approach-avoidance task was presented approximately 35 minutes after drug intake and the other half it was presented approximately 55 minutes after drug intake. At the end of testing, participants were asked to guess what substance they received, and then they were debriefed and were either offered a monetary reward, experimental credit, or chocolate.

### Statistical analysis

Independent samples t-tests were carried out to explore differences between the OT and placebo groups in baseline measures of depression, trait anxiety and attachment orientation. Furthermore, mixed model analysis of variance (ANOVA) tests were run to examine the effects of intranasal administration of drug spray (OT, placebo) and participant sex (male, female) on approach-avoidance responses to five facial emotions (anger, sadness, happiness, fear, and disgust). The effects of task order, drug expectancies and stimulus type were also examined. Post hoc Tukey HSD tests were run to explore interaction effects and Bonferroni corrections were applied to control for experiment-wise error as appropriate. All statistical analyses were performed with SPSS 16. RTs for incorrect responses (Total: 3.76%; OT: 1.9%, Placebo: 1.86%) and outliers were not included in the statistical analyses. Specifically, RTs below 300 ms and above 4000 ms were considered to be outliers [29] as were RTs 2 standard deviations above the individual mean. Three cases were also excluded because of incomplete data. To compare changes in mood between OT and placebo sessions, pre-post δ scores were calculated: Change in mood (δ)  =  Mood at Time 1 – Mood at Time 2 (Time 1: pre drug administration, Time 2: thirty minutes post drug administration). Finally, a multivariate ANOVA (MANOVA) was run on δ scores in mood with drug as between-subjects variable. Partial eta-squared values (η_p_^2^) are reported as measures of estimated effect sizes.

## Results

Independent samples t-tests showed no difference between drug groups in baseline measures of a) depression: *t*(118)  = −1.87, *p* = .06 (OT: M = 9.57, SD  = 5.47, Placebo: M = 11.62, SD  = 6.49), b) trait anxiety: *t*(118)  = −1.94, *p* = .06 (OT: M = 38.95, SD  = 10.38; Placebo: M = 42.58, SD  = 10.19), c) attachment avoidance *t*(115)  = −.94, *p* = .35 (OT: M = 34.59, SD  = 15.35, Placebo: M = 37.37, SD  = 16.73), and d) attachment anxiety, *t*(115)  = −1.86, *p* = .07 (OT: M = 35.31, SD  = 15.28, Placebo: M = 41.11, SD  = 18.29).

**Figure 1:**
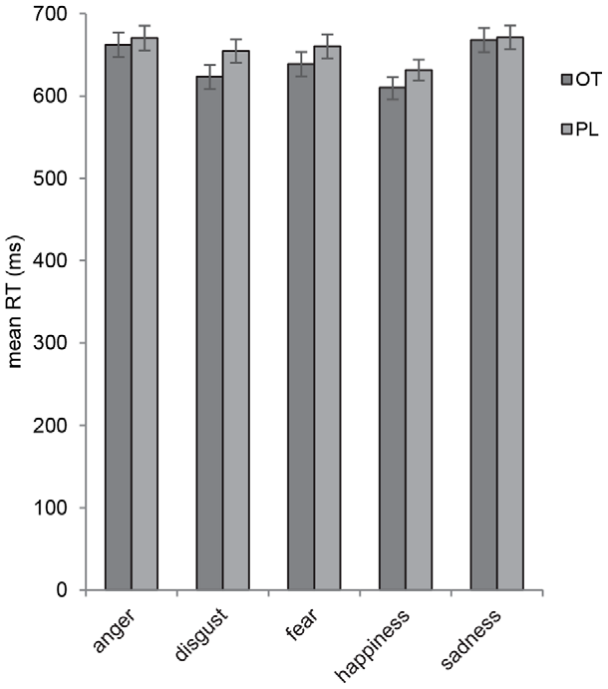
Mean reaction times to emotional face under OT and PL. Mean reaction times approaching and avoiding faces that depict anger, fear, disgust, happiness and sadness are shown for the oxytocin (OT) and placebo (PL) groups. The OT group was faster to approach and avoid faces expressing disgust compared to PL. No effect of OT was observed on approach-avoidance reactions to the other emotional expressions. Error bars show standard errors of the means.

A mixed model ANOVA was carried out on the mean RT data, with drug (OT, placebo) and participant sex (male, female) as between-subjects variables and emotion (angry, fearful, happy, disgusted, and sad), and lever direction (pull, push) as repeated measures. No main effect of drug, F(1, 113)  = .79, p = .38, or participant sex, F(1, 113)  = 1.78, p = .19, was found but the interaction between drug and participant sex was shown to be significant, F(1, 113)  = 4.23, p = .04, η_p_^2^ = .036. There was a main effect of emotion, F(3.33, 376.08)  = 37.84, p<.001, η_p_^2^ = .50, an emotion by drug interaction, F(3.33, 376.08)  = 3.00, p = .03, η_p_^2^ = .119 (Fig. 1), and an emotion by lever direction interaction, F(3.86, 436.26)  = 6.10, p<.001, η_p_^2^ = .185 (see Table 1).

**Table 1:** Mean RTs (and standard deviations) pushing and pulling a lever in response to emotional faces.

	Pull		Push	
Emotion	M	SD	M	SD
**Anger**	672.50	123.11	661.47	117.83
**Disgust**	643.37	119.94	635.87	109.96
**Fear**	649.34	130.23	651.25	110.14
**Happiness**	607.15***a***	104.16	635.60***a***	104.13
**Sadness**	668.81	121.58	671.57	115.67

M =  mean; SD  =  standard deviation; df  = 113; Same letters denote significant differences between means.

To examine the drug by participant sex interaction, univariate ANOVA tests were conducted separately for the OT and placebo groups and showed that in the OT group, females were faster than males, F(1, 56)  = 5.66, p = .02, η_p_^2^ = .092 (Females: M = 607.49, SD  = 74.42; Males: M = 671.54, SD  = 122.97), while in the placebo group, females had comparable RTs with males, F(1, 57)  = .28, p = .59 (Females: M = 664.27, SD  = 105.94; Males: M = 649.88, SD  = 103.12). Bonferroni corrections were applied here and showed that the difference in RTs between males and females in the OT group remained significant. Post hoc Tukey's tests were run to explore the emotion by drug interaction and showed that mean RTs to disgusted faces were faster for the OT group compared to placebo, p<.05, whereas the mean RTs for the remaining emotions did not differ between the two drug groups. Tukey's tests also showed that in the OT group mean RTs to angry faces were slower compared to disgusted faces, and mean RTs to sad faces were slower compared to happy faces, p values <.05, while in the placebo group, mean RTs to happy faces were faster compared to angry and sad faces, p values <.05. The main effect of emotion was not investigated as it was subsumed in two-way interactions. Furthermore, the emotion by lever direction was explored through Tukey's tests which showed that approach to happy faces was faster than avoidance (p<.05) but no differences were found in approach-avoid tendencies to all other emotions (all ps >.05). Further ANOVA tests indicated that task order and drug expectancies did not influence the effect of drug on approach-avoidance tendencies, and no differential effect of OT was found on responses to social vs. non-social stimuli, while negative stimuli elicited faster avoidance than approach responses. Stimulus type (social, non-social) and stimulus valence (negative, positive) were entered in the original ANOVA as within-subjects factors. No main effect of drug, F(1,113)  = .74, p = .39, and no significant drug × stimulus type interactions, all ps >.31, were found. There was a main effect of valence, F(1,113)  = 81.96, p<.001, η_p_^2^ = .42, with positive stimuli eliciting faster RTs compared to negative, a main effect of stimulus type, F(1,113)  = 357.13, p<.001, η_p_^2^ = .76, with social stimuli eliciting faster RTs than non-social stimuli, a stimulus type by participant sex interaction, F(1,113)  = 7.72, p = .006, η_p_^2^ = .06, a valence by lever direction interaction, F(1,113)  = 12.63, p = .001, η_p_^2^ = .10, and a stimulus type by lever direction interaction, F(1,113)  = 8.45, p = .004, η_p_^2^ = .07. Post hoc Tukey's tests showed that a) non-social stimuli elicited slower responses by males than females (p<.05), b) participants were faster to avoid than approach negative stimuli (p<.05), and c) social stimuli elicited faster responses than non-social stimuli (p<.05).

Finally, pre-post δ scores were calculated to compare changes in mood between OT and placebo sessions. A MANOVA was then run on the δ scores to examine the effect of drug and found no effect on change in affect, F(1, 115)  = .002, p = .96, wakefulness, F(1, 115)  = 05, p = .82, and calmness, F(1, 115)  = .49, p = .49.

## Discussion

The present study set out to examine whether intranasal administration of OT spray affects approach-avoidance motor responses to social (e.g., facial expressions of disgust, anger, fear, happiness and sadness) versus non-social stimuli (e.g., landscapes). To our knowledge, this is the first direct examination of the hypothesis that intranasal OT increases approach and decreases avoidance toward social vs non-social stimuli [21]. Results showed that overall RTs (across lever direction and drug treatment group) were faster in response to social versus non-social stimuli as well as positive versus negative stimuli, which is in line with a number of studies showing that positive and social stimuli are processed preferentially [40], [41], [42]. Consistent also with previous approach-avoidance motor response studies using both lexical and facial stimuli, we found that happy faces elicited faster approach than avoidance whereas negative stimuli (both social and non-social) triggered faster avoidance than approach responses [27], [29], [31], [33]. It seems therefore that on a basic level of affective processing and irrespective of drug-group allocation, positively-valenced stimuli were perceived as appetitive while negatively-valenced stimuli were perceived as aversive and led to corresponding approach/avoidance motoric responses, which is in line with previous research [43], [28], [29], [44]. These findings support the validity of the present task and give us confidence in our findings. While anger and fear can elicit both approach (fight) and avoidance (flee) tendencies [31], happy faces are the least ambiguous among the emotional expressions, explaining why they elicit the most consistent response in perceivers. Additionally, no significant changes were found in mood (i.e., affect, wakefulness, and calmness) as a result of administration of nasal OT spray, and there were no effects of task order and drug expectancy.

Our most important and novel finding was that intranasal OT administration did not enhance approach (or decrease avoidance) responses to facial expressions of anger, fear, sadness, or happiness, relative to placebo and non-social stimuli, thus failing to support the theory that OT increases approach to social stimuli and decreases avoidance [21]. Instead, the finding implies that intranasal administration of OT promotes enhanced social cognition through an alternative mechanism. This finding accords with a very recent study that failed to find a uniform effect of OT administration on approach behaviour as measured by intimacy equilibrium (i.e., increased disclosure and eye-contact) between two strangers [45]. In further agreement with our findings, the latter study reported a selective facilitative effect of oxytocin administration on disclosure in females but not males. Nevertheless, one should consider the possibility that the present null findings were observed because OT administration (at least the dosage used herein) may not have reached relevant sites of action in the brain or may not have activated an alternative pathway that would have elicited these actions. While a large number of previous studies have observed effects of intranasal administration of OT on social cognition/behavior using the same dosage as in our study (i.e., 24 IU), other studies have reported behavioral effects using higher dosages (e.g., 30 IU) making interpretation and comparison of results somewhat difficult [46]. In other words, the optimal dosage for certain effects of OT nasal spray is still undetermined.

Failure to support the approach-avoidance theory in the present study may also be due to the lack of such an effect in the context of the processing of emotional faces. Arguably, one should not discount the likelihood that the stimuli used here were simply not able to elicit social feelings or negative affect due to their lack of social animation. Perhaps the use of paradigms measuring approach-avoidance to actual people would elicit the anticipated social effect of OT on approach-avoidance responses. Simply put, an effect of OT may be stronger and hence more evident in more interactive, real-life contexts, although it should be noted that the simple perception of affective stimuli has been sufficient to induce motivational tendencies in a large number of published studies [27], [31], [32]. The facial stimuli we used here came from the Karolinska Directed Emotional Faces set [37]. This validated stimulus set [47] has been used in numerous social cognitive reaction time studies [48], [5], [49] making it unlikely that it would fail to elicit approach and avoidance in a task such as the one used herein.

A related and unexpected but nevertheless interesting finding is that compared to placebo intranasal OT administration led to faster motor responses to faces depicting disgust – regardless of lever direction, suggesting maybe that while intranasal OT did not increase approach or indeed avoidance to disgust facial expressions, it may have speeded processing of these faces. Approach-avoidance responses to facial depictions of disgust are complex, suggesting that while individuals show conscious (self-reported) avoidance of disgust they do not show automatic (motor) avoidance in an approach-avoidance task [33]. The increased salience of cues that imply threat of contagion is likely to indicate an adaptive mechanism and a quick response to displays of disgust could render an individual more prepared to protect the self (and potentially young dependents) than otherwise. We tentatively speculate that OT may be playing a prophylactic role against contamination and pathogen threat to increase chances of survival. This idea has some support from studies in human and non-human mammals showing OT administration to be involved in the detection, interpretation and modulation of responses to social cues that signal threat of contagion (for review see [50]). Importantly, the amygdala, a brain area that has been implicated in the processing of social cues that denote threat [51], is also rich in OT receptors and its activity here tends to be reduced by OT administration upon exposure to threatening stimuli [18], [16], [17]. It is likely that OT also affects amygdala activation during the processing of cues that signal the threat of possible contagion. As a consequence, OT administration may lead to faster responses to disgust depicting stimuli to counter the dangers associated with contact with contagious conspecifics. These speculative hypotheses deserve further investigation.

Our data also speak against a general social salience role for OT – at least when it comes to a facilitation of responses toward emotional facial expressions [52], [6] – as there was no evidence that emotional faces per se were more salient relative to non-social stimuli in the OT group compared to control. It is likely that the failure to find a differential effect of nasal OT spray administration on social versus non-social stimuli indicates that OT does not promote salience of social cues indiscriminately but instead may have a more nuanced effect for certain social stimuli. This conclusion may need further support in light of the fact that the number of non-social stimuli used in the present study was lower compared to that of social stimuli. This imbalance is potentially a weakness of our design, but we felt that including more non-social stimuli would make the task very long causing fatigue and boredom in participants. Future research should perhaps include equal numbers of facial and non-facial stimuli.

Nevertheless, it is worth noting here that we did observe other effects of OT administration that lead us to believe that the present dosage was sufficient for at least specific behavioral effects of OT nasal spray. For example, results showed that females receiving OT nasal spray were faster overall than males, whereas females receiving placebo had comparable reaction times with their male counterparts supporting the idea that OT may play a more important role in female than male behavior [53]. Indeed, this finding fits with the crucial function of OT as a female hormone in labor, bonding and parenting both in human- [54], [4] and non-human mammals [55] and is in agreement with recent experimental evidence pointing to the sex-dimorphic effects of OT nasal spray in humans [19], [45] [56], [57].

Importantly, we measured a number of psychological variables at baseline to account for the contribution of relevant individual differences in the effects of OT nasal spray, as has been argued necessary by other OT researchers [52]. No differences were found between drug groups with regard to trait anxiety, depression, attachment avoidance and attachment anxiety, indicating that these baseline traits did not influence the above findings.

### Limitations

While previous research strongly suggests that intranasal administration of OT can reliably influence socially relevant judgments and ratings, relative to placebo [58], [17], [5], findings from studies using lower level tasks administered after OT ingestion (RTs to emotion stimuli and emotion recognition tasks, for example) tend to be mixed and sometimes inconsistent [13], [10], [14], [11], [12]. On this basis, our findings need replication to allow confidence in the pattern of results. Furthermore, our exclusive use of male facial stimuli potentially weakens the generalizability of our findings. However, it is worth noting that previous studies have shown that the enhancing effect of OT nasal spray on the processing of faces is independent of the sex of stimuli faces [10], [34], [12].

A further potential limitation of our study concerns the unequal number of negative and positive emotional faces. Admittedly, the negative facial stimuli presented here outnumber the positive ones but this is inevitable given that there is only one basic positive emotion, namely, happiness – at least as found in validated and established facial expression data sets designed for experimental purposes. Additionally, while it is possible that the presentation of unequal numbers of negative versus positive stimuli could bias the effect of OT on the avoidance of negative stimuli and the approach of positive stimuli, our findings did not indicate the presence of such a bias.

Furthermore, although the scenes do not necessarily match the facial expressions of emotion finding a perfect scene match for a face represents a near-impossible task. We feel that scenes that are devoid of facial images are appropriate non-social stimuli. Indeed, previous published OT studies have also compared images of landscapes to faces [16], [34].

## Conclusion

In conclusion, our results suggest that intranasal administration of OT does not appear to increase approach or decrease avoidance toward social stimuli, in this case faces depicting various emotions. Our data fail also to support the social salience hypothesis of OT's effects on social cognition but tentatively suggest a prophylactic function role for OT, as reflected in faster responses to affective stimuli that signal the risk of contagion. Being able to respond quickly to potential contagion in one's environment is adaptive. To further examine this proposal, future research could examine the impact of OT on responses to faces that display signs of disease as these suggest contamination.

Future research could examine the impact of nasal OT spray administration in real-life settings whereby actual approach-avoidance behaviour towards a confederate could be examined. Given the well-documented role of OT in social behaviour it may be that it would have a more pronounced effect on approach-avoidance tendencies in a setting where interaction with real people is involved [45]. Despite yielding no support for either of the two dominant theories that have been put forward to account for OT's role in social cognition, the current study is nevertheless informative regarding the function of OT in social cognition and specifically how OT spray may and may *not* affect the processing of socially-relevant cues. As such the present findings are a valuable addition to extant knowledge in the young field of OT research.
